# Therapy Efficacy in Chronic Aphasia

**DOI:** 10.3233/BEN-2011-0342

**Published:** 2011-11-07

**Authors:** Anna Basso, Margherita Macis

**Affiliations:** ^1^Department of Neurological SciencesMilan UniversityMilanItaly; ^2^Faculty of PsychologyUniversity of Milano-BicoccaMilanItaly

**Keywords:** Chronic aphasia, intensive/prolonged treatment, recovery

## Abstract

There is good evidence that aphasia therapy is effective if sufficiently prolonged or intensive and that chronic aphasic individuals can also benefit from therapy, but data on chronic aphasia are scanty.

The aim of this retrospective study was to investigate whether chronic aphasia benefits from a very intensive therapeutic regimen. We revised the files (January 2000 to December 2008) of the chronic subjects whom we suggested have periodic sessions in our Unit (generally once a week) and 2–3 hours daily of homework with the help of a family member, supervised and controlled by the speech-therapist. Treatment would go on as long as amelioration is evident. Results for 23 chronic aphasic subjects are reported. All subjects had undergone previous therapy and 10 had been dismissed because no further recovery was expected. Recovery was significant in oral and written nouns and actions naming, oral and written sentence production and Token Test scores. Only 4 subjects did not improve. Severity of the disorder did not predict success or failure. We conclude that recovery was due to the intense work done. Further, we believe such a regimen could be successful in a number of patients for whom a less intensive regimen would not be effective.

## 1. Introduction

Not everyone would agree that aphasia therapy is effective. Schoonen, for instance, wrote "How can we explain the "significant" improvement in so many studies? One of the "explanations" is that these researchers were just lucky [33]." Experimental evidence, though not definitive, is clearly in favor of aphasia therapy efficacy. Four meta-analyses [30–32,37] and two evidence-based reviews [9–12] concluded that treated aphasic subjects improve more than untreated subjects. Recently, the Cochrane review [16] came to a positive but more doubtful conclusion: "Significant differences between the groups' scores were few but there was some indication of a consistency in the direction of results which favoured the provision of SLT".

Not all studies that compared treated and untreated subjects came to the same positive conclusion; Pickersgill and Lincoln [26], for example, found no differences between treated and untreated subjects, and David et al. [13] and Lincoln et al. [18] found no significant difference in amount of recovery between subjects treated by therapists and subjects treated by volunteers. These negative results only appear to contradict the statement that aphasia therapy is effective. Bhogal et al. [7] compared intensity and quantity of therapy in positive and negative studies whose target outcome measures were the Token Test [14], the Porch Index of Communicative Abilities [27], and the Functional Communication Profile [15]. Eight studies (four positive and four negative) provided sufficient data to allow determination of the relationship between results and intensity/quantity of treatment. The total number of treatment hours in the positive studies was 98.4 and in the negative studies 43.6; the difference was significant. In effect, an insufficient amount of treatment is not efficacious. Confirmation of the importance of treatment quantity comes from a meta-analysis [31]. Intensity of treatment has also been proved to be an important factor in recovery and groups of chronic aphasic individuals who underwent intensive training based on communicative language games (CIAT) showed positive results [21,22, 28].

A thorny question for the study of aphasia therapy efficacy is spontaneous recovery, which is present in

ISSN 0953-4180/11/$27.50 © 2011 – IOS Press and the authors. All rights reserved almost all aphasic individuals in the first months post-stroke, even if it may be very limited. Some authors [1, 8,19] report some positive results in group of chronic subjects who started treatment after the period of spontaneous recovery but more interesting are the results of a research by Moss and Nicholas [25]. They re-analyzed data from 23 studies for a total of 57 subjects, subdivided in 6 groups according to time-post-onset (TPO) – 1 to 6 years – and compared the effect of TPO on recovery. No significant difference in amount of recovery among the 6 groups was found.

The potential effect of other therapeutic strategies has recently been explored. Current research on biological approaches, such as pharmacotherapy and non-invasive cortical stimulation methods provides encouraging data [6,35]. Glutamatergic, monoaminergic, and cholinergic drugs [29] and Transcranial Magnetic Stimulation and Direct Current Stimulation [e.g. [23]] have been shown to have a positive effect on either acute or chronic aphasic individuals' performance in language tasks

In summary, there is good evidence that aphasia therapy is effective if sufficiently prolonged or intensive and that chronic aphasic individuals can also benefit from therapy. Moreover, the combined use of biological and behavioral treatment approaches seems an interesting prospective to enhance recovery [5,20].

The amount of therapy suggested to aphasic subjects at the Aphasia Unit of Milan University has increased steadily during the years. In the last years it has reached very high standards, requiring great efforts on the part of the aphasic subjects and their families. The regimen we suggest consists of periodic sessions in our Unit (generally once a week for aphasic individuals living in Milan or nearby and once or twice per month for those living far from Milan) and intensive homework (2–3 hours per day) supervised by the speech therapist; treatment is considered to be concluded when no recovery is observable between two control evaluations, 3 to 6 months apart. This regimen in not easy to implement; we must persuade aphasic individuals and their family that their convinced commitment is necessary for treatment to be efficacious, and find a way to implement the necessary homework with the help of a relative, a friend or a volunteer.

In 2001, Basso and Caporali [4] checked whether this regimen was effectively better than a more standard regimen of 5 sessions per week. Results of 3 pairs of vascular aphasic subjects comparable for sex, age, education, TPO, site and size of lesion and type of aphasia were compared. Control subjects had been treated 1-hour daily and the experimental subjects, besides daily sessions, had underwent 2–3 hours daily treatment at home with the aid of a family member. All subjects were treated for as long as some recovery was evident. All patients improved but experimental subjects improved more and, more importantly and contrary to the control subjects, used their recovered language in daily living.

Those were only 3 subjects but we were interested in knowing whether the results of such a regimen are really worth the effort and we decided that a more comprehensive study was necessary. In this paper we consider all the chronic subjects seen between January 2000 (when we regularly started to propose an intensive treatment) and December 2008 whom we suggested a similar regimen.

## 2. Subjects

In the period January 2000 – December 2008, 443 vascular subjects were evaluated at the Aphasia Unit of Milan University. Intensive/prolonged treatment was not suggested to 337 subjects for the following reasons: over 75 of age (56), with an incomplete evaluation (31), not or only mildly aphasic (148), in rehabilitation elsewhere (45), for other reasons (57).

Of the remaining 106 individuals to whom an intensive/prolonged treatment was suggested, 62 were acute or sub-acute (less than 6 MPO) and do not concern us here. All 44 chronic subjects (range 7-106 MPO) had undergone aphasia therapy (range 2–71 months) in different hospitals before being evaluated at the Aphasia Unit and the option to resume treatment was proposed to them. When resumption of treatment with an intensive regimen was proposed, 12 potential subjects (Group 1) refused, and an additional 9 (Group 2) abandoned treatment. Thus, the treatment group comprised 23 chronic subjects (Group 3). These persons were treated for at least 6 months.

Table 1 reports demographical and clinical data for these 23 subjects who completed at least 6 months of intensive therapy (range: 6–60). Nine had global aphasia, 3 mixed non-fluent, 6 Broca and 5 severe Wernicke aphasia. It will be remembered that all had already been treated (range 2–50 months) and 10 had been dismissed because no further recovery was expected.

Table 1
Demographic and clinical data of the 23 subjects treated for at least 6 months (Group 3)

| Subject | Sex | Age | Education (years) | MPO | Previous therapy (months) | Token Test (0–36) | Aphasia type |
|---|---|---|---|---|---|---|---|
| 5141 | F | 32 | 17 | 31 | 24 | 8 | G |
| 5170 | M | 54 | 16 | 7 | 6# | 2 | W |
| 5203 | M | 75 | 17 | 9 | 8 | 9 | W |
| 5204 | M | 74 | 17 | 8 | 7 | 13 | G |
| 5223 | F | 41 | 8 | 64 | 50# | 19 | B |
| 5237 | F | 26 | 13 | 63 | 36# | 26 | B |
| 5250 | F | 67 | 13 | 7 | 6 | 9 | G |
| 5253 | M | 38 | 13 | 7 | 3 | 8 | G |
| 5298 | M | 59 | 12 | 9 | 8# | 18 | MNF |
| 5302 | M | 51 | 11 | 9 | 8# | 0 | G |
| 5334 | M | 66 | 13 | 16 | 13# | 13 | G |
| 5348 | M | 44 | 13 | 17 | 7# | ° | G |
| 5406 | M | 42 | 8 | 25 | 2 | 20 | B |
| 5432 | F | 52 | 13 | 18 | 14 | 13 | W |
| 5452 | M | 48 | 13 | 11 | 9 | 1 | W |
| 5469 | F | 50 | 17 | 30 | 10# | 18 | B |
| 5475 | F | 66 | 8 | 12 | 6 | 2 | G |
| 5504 | F | 49 | 8 | 45 | 17 | 16 | B |
| 5508 | M | 66 | 5 | 11 | 5# | 6 | G |
| 5527 | F | 54 | 17 | 10 | 7 | 25 | B |
| 5599 | M | 43 | 8 | 13 | 3# | 17 | MNF |
| 5600 | M | 65 | 13 | 10 | 6 | 17 | MNF |
| 5602 | M | 68 | 17 | 12 | 5 | 2 | W |

Legend: F = Female, M = Male, G = Global, W = Wernicke, B = Broca, MNF = Mixed Non Fluent, ° = Colour blind subject, # = Dismissed because it was considered that treatment was no longer effective.

## 3. Treatment

Except the 6 Broca aphasic individuals, all subjects were severely aphasic and aim of the treatment was to rebuild the aphasic individuals' capacity to sustain a simple conversation in daily living. The initial treatment goals were: a) improve understanding of conversational roles, b) improve comprehension of the message in "ecological" situations, including comprehension of the speaker's intention (whether it is a request of information, a communication, or a request to do something), and c) have the subjects produce more correct and longer responses.

To improve understanding of conversational roles, the clinician must make clear that she and the aphasic individual are supposed to do something in turn. Initially, this can be achieved by having the aphasic partner act in any way independently of the clinician's request, as long as this sets up a continuous exchange with the clinician. When role-switching is restored, the next step is for the clinician to make herself understood. To facilitate the hearer's job she must use some expedients: a) keep eye contact, b) make clear which speech act (ask a question, state something, or solicit the patient to do something) she is going to do by making it explicit, c) help the aphasic patient recognize what the question is about by placing the topic of the sentence at the beginning ("London, have you ever been there?" rather than "Have you ever been in London?"), d) use all communication channels (verbal, gestural, writing, drawing, mimic), and e) verbalise the message in the simplest possible way. Questions must obviously require only short and easy responses and any response given by the aphasic subject, independently of whether it is wrong or apparently unrelated to the question posed, must be accepted and reacted to as if it were correct and related to the question. When the aphasic individual is totally unable to give a response, the clinician must offer the response for which she presumes the subject is aiming and, if he agrees, have the patient repeat it. After presenting the same question again, she poses different questions that involve the same response because it is presumable that producing a word in conversation repeatedly would facilitate its future retrieval [3]. Besides these common goals, for each patient specific goals – such as reduction of apraxia of speech, prevention of agrammatism, recovery of word finding abilities, reading aloud – were also identified.

Table 2
Comparison of mean age, education, MPO, previous therapy (months) and Token Test scores of Group 1 ($n = 12$) and Group 2 ($n = 9$) subjects

| | *Group 1* | | *Group 2* | | *T test* |
|---|---|---|---|---|---|
| | Mean | SD | Mean | SD | p value |
| Age | 56.3 | 12.9 | 58.2 | 15 | 0.760 |
| Education (years) | 10.8 | 3.7 | 12.1 | 3.2 | 0.391 |
| MPO | 33.7 | 22.4 | 29.9 | 32.3 | 0.754 |
| Previous therapy (months) | 18.3 | 18.2 | 11.8 | 5.3 | 0.311 |
| Token Test (0–36) | 11.8 | 8.8 | 8.8 | 8.7 | 0.452 |

Table 3
Comparison of demographic data and Token Test scores of Groups 1+2 ($n = 21$) and Group 3 ($n = 23$)

| | Group 1+2 | | Group 3 | | T test |
|---|---|---|---|---|---|
| | Mean | SD | Mean | SD | p value |
| Age | 57.1 | 13.5 | 53.5 | 13.4 | 0.371 |
| Education (years) | 11.3 | 3.5 | 12.6 | 3.7 | 0.245 |
| MPO | 32 | 26.4 | 19.3 | 16.8 | 0.061 |
| Previous therapy (months) | 15.5 | 14.3 | 11.3 | 11.3 | 0.282 |
| Token Test scores (0–36) | 10.5 | 8.7 | 11.9 | 7.8 | 0.571 |

The therapeutic program was more heterogeneous for the 6 Broca aphasic subjects. Four, with reduced speech and agrammatism, were asked to identify action verbs in a vocabulary, read its definition, write it down and repeat it aloud; look up in the dictionary for another action verb, repeat the whole procedure and then try to retrieve the previously rehearsed verbs. The family "therapist" had to explain the patient the structure of the verb (to kill, for instance, requires two arguments, both animate, the killer and the one killed) and ask the patient to produce a sentence with the verb and all the obligatory complements. In short, the family "therapist" were required to deliver a simplified mapping therapy [2,24]. For the two Broca aphasic that did not present agrammatism, therapy was approximately the same without, however, any particular attention on the structure of action verbs.

Tasks that could easily be explained and transferred to a naïve person or could be performed independently by the aphasic individuals were chosen and explained to the aphasic subjects and their family "therapist" and their therapeutic behavior were regularly supervised. When the therapeutic relationship between the aphasic person and their care-givers was well established, they were asked to spend 3–4 consecutive days at the Aphasia Unit and treatment of conversation was explained and shown to the care-givers; their behavior in this task was supervised.

Subjects were asked to work regularly 2–3 hours per day (partly on their own and partly with the family member) and records of their home-work was reported in our files. The frequency of their visits to the Aphasia Unit (from once a week to once a month, each time for 2–3 hours) depended on two main factors: how far they lived from Milan and the complexity of their disorders.

## 4. Results

Groups 1 and 2 did not differ significantly for age, education, MPO, length of therapy underwent prior to our examination or severity of aphasia as evaluated by the Token Test (Table 2).

We pooled together Groups 1 and 2 subjects and compared these variables to those of Group 3. None of the difference was significant (Table 3).

Table 4 reports percentage of correct responses in oral and written noun and action naming and picture description, and Token Test's scores at first and last evaluation of the 23 treated subjects.

Mean scores at first and last evaluation were compared by t test for paired samples. All differences were significant (Table 5 and Fig. 1).

All subjects were chronic and all had been previously treated in other hospitals and it is very unlikely that their gains could be explained by spontaneous recovery or other unspecified factors. The most obvious explanation is that amelioration was the result of treatment. However, we compared recovery in oral and written tasks because improvement in oral tasks is likely to be more sensitive to unspecific use of language in daily living but written language is not frequently used in daily living, especially by aphasic subjects and amelio-

Table 4
Percentage of correct responses in oral and written noun and action naming, sentence production, and Token Test's scores at first and last evaluation of treated subjects

| | Noun naming (0–40) | | | | Action naming (0–20) | | | | Sentence production (0–20) | | | | Token Test (0–36) | |
|---|---|---|---|---|---|---|---|---|---|---|---|---|---|---|
| | *Oral* | | *Written* | | *Oral* | | *Written* | | *Oral* | | *Written* | | | |
| Subject | First | Last | First | Last | First | Last | First | Last | First | Last | First | Last | First | Last |
| 5141 | 60 | 80 | 7.5 | 75 | 50 | 60 | 0 | 50 | 10 | 30 | 0 | 25 | 8 | 12 |
| 5170 | 0 | 32.5 | 0 | 25 | 0 | 40 | 0 | 0 | 0 | 30 | 0 | 0 | 2 | 13 |
| 5203 | 85 | 87.5 | 97.5 | 95 | 100 | 80 | 100 | 85 | 60 | 70 | 90 | 85 | 9 | 18 |
| 5204 | 0 | 15 | 0 | 10 | 0 | 35 | 0 | 0 | 0 | 5 | 0 | 0 | 13 | 23 |
| 5223 | 80 | 87.5 | 50 | 60 | 80 | 70 | 55 | 45 | 40 | 60 | na | na | 19 | 20 |
| 5237 | 97.5 | 97.5 | 87.5 | 87.5 | 90 | 95 | 70 | 85 | 90 | 90 | 90 | 100 | 26 | 27 |
| 5250 | 0 | 62.5 | 0 | 40 | 0 | 35 | 0 | 10 | 0 | 10 | 0 | 0 | 9 | 25 |
| 5253 | 32.5 | 55 | 0 | 5 | 15 | 30 | 0 | 5 | 0 | 30 | 0 | 0 | 8 | 20 |
| 5298 | 0 | 40 | 0 | 30 | 0 | 20 | 0 | 0 | 0 | 5 | 0 | 0 | 18 | 18 |
| 5302 | 0 | 15 | 0 | 0 | 0 | 5 | 0 | 0 | 0 | 0 | 0 | 0 | 0 | 3 |
| 5334 | 50 | 92.5 | 10 | 75 | 30 | 95 | 0 | 50 | 10 | 60 | 0 | 65 | 13 | 20 |
| 5348 | 0 | 7.5 | 22.5 | 55 | 0 | 0 | 10 | 15 | 0 | 0 | 0 | 25 | ° | ° |
| 5406 | 75 | 75 | 35 | 35 | 40 | 95 | 0 | 10 | 30 | 20 | 0 | 45 | 20 | 26 |
| 5432 | 2.5 | 82.5 | 5 | 82.5 | 30 | 80 | 0 | 80 | 20 | 60 | 0 | 80 | 13 | 19 |
| 5452 | 37.5 | 85 | 0 | 40 | 65 | 90 | 0 | 35 | 10 | 60 | na | na | 1 | 14 |
| 5469 | 37.5 | 87.5 | 30 | 90 | 35 | 70 | 20 | 80 | 0 | 90 | 0 | 90 | 18 | 25 |
| 5475 | 0 | 37.5 | 0 | 12.5 | 0 | 0 | 0 | 0 | 0 | 0 | 0 | 0 | 2 | 10 |
| 5504 | 70 | 87.5 | 55 | 90 | 60 | 85 | 25 | 85 | 50 | 90 | 35 | 70 | 16 | 29 |
| 5508 | 0 | 0 | 0 | 5 | 0 | 0 | 0 | 0 | 0 | 0 | 0 | 0 | 6 | 10 |
| 5527 | 52.5 | 82.5 | 62.5 | 95 | 45 | 85 | 45 | 90 | 80 | 100 | 85 | 100 | 25 | 28 |
| 5599 | 10 | 50 | 5 | 15 | 0 | 40 | 0 | 5 | 0 | 15 | 0 | 5 | 17 | 27 |
| 5600 | 20 | 70 | 7.5 | 65 | 50 | 75 | 0 | 80 | 0 | 70 | 0 | 50 | 17 | 23 |
| 5602 | 32.5 | 70 | 0 | 50 | 50 | 60 | 0 | 60 | 0 | 70 | 0 | 25 | 2 | 6 |

Legend: na = Not administered, ° = Colour blind subject.

Table 5
Comparison of oral and written noun and action naming, sentence production, and Token Test mean scores at first and last evaluation

| Task | Modality | First evaluation | | Last evaluation | | T test |
|---|---|---|---|---|---|---|
| | | Mean | SD | Mean | SD | (p value) |
| Noun naming | Oral | 12.9 | 13.2 | 24.3 | 12.1 | $< 0.0001$ |
| | Written | 8.3 | 12 | 19.8 | 13.1 | $< 0.0001$ |
| Action naming | Oral | 6.4 | 6.5 | 10.8 | 6.7 | $< 0.0001$ |
| | Written | 2.8 | 5.5 | 7.6 | 7.2 | 0.001 |
| Sentence production | Oral | 3.5 | 5.5 | 8.4 | 6.9 | 0.0002 |
| | Written ($n = 21$) | 2.9 | 6.4 | 7.3 | 7.7 | 0.002 |
| Token Test ($n = 22$) | | 11.9 | 7.8 | 18.9 | 7.4 | $< 0.0001$ |

ration in written tasks is most probably due to therapy. None of the differences was significant (Table 6).

We also considered severity of aphasia as a possible prognostic factor of recovery. We divided treated subjects in two subgroups (with Token Test's scores under or above the mean of the group) and compared their gains in the Token Test (Table 7). Token Test's scores of the "severe" subgroup were significantly lower at first and last examination but the difference between the 2 scores was not significantly different in the "severe" and "moderate" subgroups.

Particularly interesting appears to be recovery in picture description because of the use of sentences. We report two examples. Subject 5602, a 68 year-old, lawyer who suffered a CVA in the left hemisphere 12 months before being evaluated at the Aphasia Unit had undergone 5 months therapy in Florence and had been dismissed because no further recovery was expected. His spontaneous speech consisted in a sort of mixed jargon (verbal paraphasias and neologisms), totally incomprehensible. Seven months later he described a picture of a living room in which a woman is knitting, a man sits in an armchair reading a newspaper, a young girl is watching television, a boy is playing with cubes, and a cat is playing with a ball of wool, as follows:

"The small cat with the small ball to play; they play

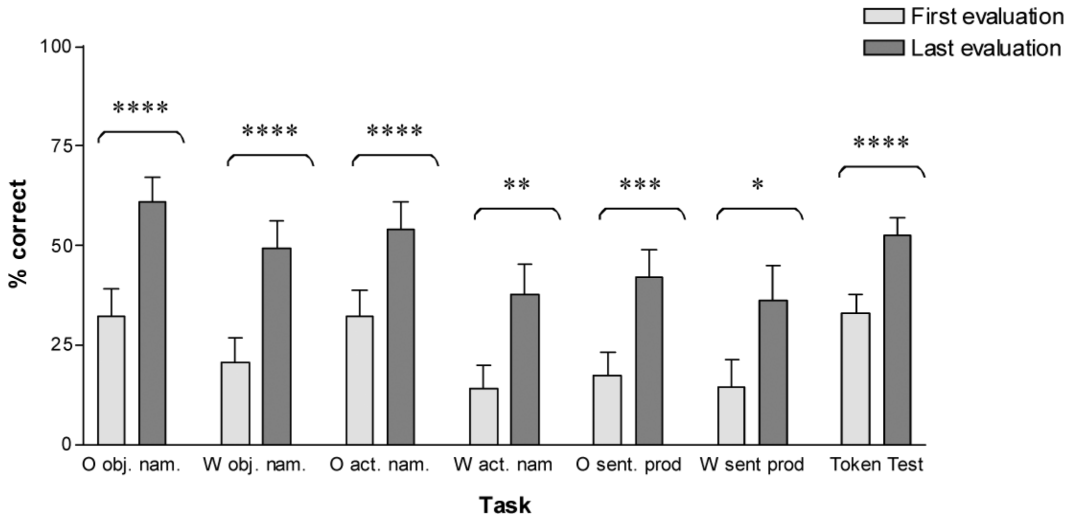

Fig. 1. Comparison of mean scores (%) in oral noun naming, written noun naming, oral action naming, written action naming, oral sentence production, written sentence production and Token Test scores at first and last evaluation of the 23 treated subjects. Error bars are SEM (standard error of the mean). Legend: O = Oral, W = Written, obj. nam = Noun naming, act. nam. = Action naming, sent. prod. = Sentence production, $^{*}p = 0.002$, $^{**}p = 0.001$, $^{***}p = 0.0002$, $^{****}p < 0.0001$.

with this ball with the mother to knit sweater. The girl 4–5 years old looks TV; she is the only one to look at it. The grandfather who reads the newspaper and doesn't see TV but the boy who plays with different pieces, short, long pieces."

Subject 5170 was 7 MPO when he arrived at Milan Aphasia Unit. He was 54-years-old and had become aphasic following rupture of an aneurysm. He had already been treated but after 6 months he had been dismissed because no further recovery was expected. His speech was fluent, characterized by phonemic jargon interspersed with existing words but deprived of any communicative value. Comprehension was very severely impaired. Both he and his wife were highly motivated and immediately accepted our proposal. Therapy went on for 5 years with slow but constant improvement. When they finally terminated therapy, subject 5170 described his day as follows:

"Then I eat... and then I don't know, it depends. I wanted to go with the ... with the ... (mimes cycling) with the bicycle, but now it hurts (points at his leg). Generally I go cycling and look around. I look around something here, something there, it's not like Milan. I also went along, but it was a bit painful... and then I turned and arrived to the Po. Then I come home and eat. Afterwards I look at the newspaper, what is in there, and then I go out again, not cycling I walk. I walk a little but now it hurts (points at his leg again). After I go... I look what is around... When I am at home I listen to the ... (mimes earphones), to that, do you understand? In the evening I eat and then I look (mimes TV), what's on, a bit of everything, and then I go to bed. Then everything starts again... as before."

Table 6
Comparison of recovery in oral and written tasks

| | Oral task | | Written task | | T test |
|---|---|---|---|---|---|
| | Mean | SD | Mean | SD | (p value) |
| Noun naming | 11.4 | 8.7 | 11.5 | 10 | 0.953 |
| Action naming | 4.4 | 4.3 | 4.7 | 6 | 0.770 |
| Sentence production | 4.9 | 5.4 | 4.4 | 5.7 | 0.776 |

Table 7
Comparison of Token Test scores at first and last evaluation of severe ($n = 10$) and moderate subjects ($n = 12$)

| | Severe | | Moderate | | T test |
|---|---|---|---|---|---|
| | Mean | SD | Mean | SD | (p value) |
| First | 4.7 | 3.6 | 17.9 | 4.3 | < 0.0001 |
| Last | 13.1 | 6.6 | 23.8 | 3.8 | 0.0001 |
| Difference | 8.4 | 4.6 | 5.9 | 4 | 0.175 |

Table 8 reports reasons why treatment was suspended, regularity of home-treatment, and the degree of improvement.

The most frequent reasons for suspension of treatment were either that subjects had reached a plateau and further recovery was not expected or that they were weary. Degree of recovery appears to be independent from suspension reasons. Regularity of home-treatment appears to be an important factor in recovery.

Table 8
Therapy length, suspension reasons, regularity of home-treatment and level of improvement

| | Subject | Therapy length (months) | Reason for suspension | Regularity | Level of improvement (0–3) |
|---|---|---|---|---|---|
| Dismissed | 5141 | 19 | Plateau | Irregular | 1 |
| | 5223 | 28 | Plateau | Irregular | 1 |
| | 5253 | 29 | Plateau | Regular | 3 |
| | 5334 | 29 | Plateau | Regular | 3 |
| | 5475 | 25 | Plateau | Regular | 1 |
| | 5432 | 18 | Too dependent on therapist | Irregular | 3 |
| | 5504 | 13 | Too dependent on therapist | Irregular | 3 |
| | 5298 | 11 | Don't work | Irregular | 1 |
| | 5348 | 36 | Don't work | Irregular | 0 |
| | 5527 | 7 | Don't work | Irregular | 3 |
| Self-dismissed | 5170 | 60 | Too weary | Regular | 3 |
| | 5237 | 10 | Too weary | Irregular | 0 |
| | 5302 | 24 | Too weary | Regular | 0 |
| | 5406 | 7 | Too weary | Irregular | 2 |
| | 5599 | 9 | Too weary | Regular | 2 |
| | 5600 | 6 | Too weary | Regular | 3 |
| | 5204 | 12 | Health | Regular | 1 |
| | 5250 | 20 | Health | Regular | 3 |
| | 5452 | 22 | Health | Irregular | 2 |
| | 5508 | 12 | Health | Irregular | 0 |
| | 5203 | 6 | Moved | Regular | 2 |
| Still in treatment | 5469 | 17 | | Regular | 3 |
| | 5602 | 7 | | Regular | 3 |

Legend: 0 = no improvement, 1 = little improvement, 2 = good improvement, and 3 = very good improvement.

## 5. Discussion

In summary the conclusions that can be drawn from our study are the following: 1) 50% of the subjects, even after a previous treatment, agreed to start an intensive and supposedly very long period of treatment with the help of a family member; 2) no difference was found between subjects who agreed to treatment and those who refused it in type and severity of aphasia, MPO, age, education and previous treatment; 3) as a group, treated subjects recovered significantly in all behaviors studied and severity of aphasia was not a negative prognostic factor; 4) recovery appears to be due to treatment, particularly because it was not different in oral and written tasks and it is difficult to imagine that writing ameliorate spontaneously in chronic severe aphasic individuals; 5) in all cases it was reported that they were more communicative in their daily living. Finally, although no definitive conclusion is possible, inspection of Table 8 seems to indicate a convincing relationship between regularity of home-treatment and recovery.

This is a retrospective study and its aim was clinical. The question was: do chronic aphasic individuals still benefit from therapy and do we have enough evidence that the therapeutic regimen we suggest is worth while the effort it requires? The answer can only be given by the aphasic subjects and their family but the very fact that in many cases treatment lasted more than a year can be read as a positive answer. Sometimes we had to insist to put an end to it; the aphasic subjects and their "family therapists" appear very much motivated and are prepared to go on forever as was the case with subject 5170 and his wife who went on for 5 years regularly working 2–3 hours per day, showing some improvement at each control evaluation. From our point of view the response is an unequivocal "yes", even for subjects that are considered to represent the most unlikely examples of likely recovery: subjects were chronic, most were very severely impaired, all had already been treated, and 10 had been dismissed because no further recovery was expected. Only 4 subjects did not show any improvement and as many as 10 showed a substantial improvement.

Probably the question of aphasia therapy efficacy could have been settled satisfactorily, if it were recognized that treatment regimen is an important determinant of recovery and that the main reason why therapy was not found to be efficacious in some studies that compared treated and untreated subjects [17,18,34,36] is that it was not sufficiently intensive and/or prolonged.

In efficacy studies the content of therapy has rarely been taken into account but it goes without saying that results depends on what is done and not only for how long or with what frequency it is done. Treatment was specifically devised and different for each of the 23 treated subjects but due to the severity of the disorder the goal was the same for most of them: sustain a simple conversation in daily living, conversation being the fundamental and primary type of language use. Moreover, the involvement of naïve "therapists" reduced the choices of possible tasks. It is possible that with different and more varied therapeutic interventions better results would have been achieved. What we do want to stress, however, is that recovery never showed up suddenly after a short period of treatment; amelioration was slow and continuous. Recovery, and particularly their use of language for communication purposes, was the result of the intense work done and we argue that such a regimen could be successful in a number of patients for whom a less intensive regimen would be ineffective.

Therapy cannot be delivered with such an intensity and for such a long time by speech therapists alone; it must be a mutual endeavor which sees the aphasic subjects, their families, and the speech therapists as active collaborators. Meinzer et al. [22] trained a group of family members to deliver CIAT to 10 chronic aphasic individuals and compared their results to those of 10 chronic aphasic subjects treated by experienced therapists. Both groups improved and there was no significant difference between the 2 groups.

In this perspective, speech therapists' tasks are more varied and more demanding. They have to locate the damage, decide what is the best treatment, establish which tasks aphasic subjects can carry out on their own and which with the help of a family member, show them how to perform the required tasks, regularly check what is going on between the subject and the lay person delivering therapy, perform the control evaluations, and adapt the intervention to the subject's recovery. Put differently, they are no longer the first violinists but conductors of a harmonious orchestra.

## References

[1] J.L. Aten, M.P. Caligiuri and A.L. Holland, The efficacy of functional communication therapy for chronic aphasic patients, *The Journal of Speech and Hearing Disorders* **47** (1982), 93–96.

[2] A. Basso, *Aphasia and Its Therapy*, Oxford University Press, 2003.

[3] A. Basso, Natural conversation: A treatment for severe aphasia, *Aphasiology* **24** (2010), 466–479.

[4] A. Basso and A. Caporali, Aphasia therapy or the importance of being earnest, *Aphasiology* **15** (2001), 307–332.

[5] M.L. Berthier, C. Green, J.P. Lara, C. Higueras, M.A. Barbancho, G. Dávila and F. Pulvermüller, Memantine and constraint-induced aphasia therapy in chronic poststroke aphasia, *Annals of Neurology* **65** (2009), 577–585.

[6] M.L. Berthier and F. Pulvermüller, Neuroscience insights improve neurorehabilitation of poststroke aphasia, *Nature reviews Neurology* **7** (2011), 86–97.

[7] S.K. Bhogal, R. Teasell and M. Speechley, Intensity of aphasia therapy, impact on recovery, *Stroke; a journal of cerebral circulation* **34** (2003), 987–993.

[8] H. Broida, Language therapy effects in long term aphasia, *Archives of Physical Medicine and Rehabilitation* **58** (1977), 248–253.

[9] S.F. Cappa, T. Benke, S. Clarke, B. Rossi, B. Stemmer and C.M. van Heugten, EFNS guidelines on cognitive rehabilitation: report of an EFNS task force, *European Journal of Neurology* **10** (2003), 11–23.

[10] S.F. Cappa, T. Benke, S. Clarke, B. Rossi, B. Stemmer and C.M. van Heugten, EFNS guidelines on cognitive rehabilitation: report of an EFNS task force, *European Journal of Neurology* **12** (2005), 665–680.

[11] K.D. Cicerone, C. Dahlberg, K. Kalmar, D.M. Langenbahn, J.F. Malec, T.F. Bergquist, T. Felicetti, J.T. Giacino, J.P. Harley, D.E. Harrington, J. Herzog, S. Kneipp, L. Laatsch and P.A. Morse, Evidence-based cognitive rehabilitation: recommendations for clinical practice, *Archives of Physical Medicine and Rehabilitation* **81** (2000), 1596–1615.

[12] K.D. Cicerone, C. Dahlberg, J.F. Malec, D.M. Langenbahn, T. Felicetti, S. Kneipp, W. Elmo, K. Kalmar, J.T. Giacino, J.P. Harley, L. Laatsch, P.A. Morse and J. Catanese, Evidence-based cognitive rehabilitation: updated review of the literature from 1998 through 2002, *Archives of Physical Medicine and Rehabilitation* **86** (2005), 1681–1692.

[13] R. David, P. Enderby and D. Bainton, Treatment of acquired aphasia: speech therapists and volunteers compared, *Journal of Neurology, Neurosurgery, and Psychiatry* **45** (1982), 957–961.

[14] E. De Renzi and P. Faglioni, Normative data and screening power of a shortened version of the Token Test, *Cortex* **14** (1978), 41-49.

[15] A.L. Holland, *Communicative Abilities in Daily Living*, Baltimore: University Park Press, 1980.

[16] H. Kelly, M.C. Brady and P. Enderby, Speech and language therapy for aphasia following stroke, *Cochrane Database of Systematic Reviews Online* **5** (2010), CD000425.

[17] E. Levita, Effects of speech therapy on aphasics' responses to Functional Communication Profile, *Perceptual and Motor Skills* **47** (1978), 151–154.

[18] N.B. Lincoln, E. McGuirk, G.P. Mulley, W. Lendrem, A.C. Jones and J.R. Mitchell, Effectiveness of speech therapy for aphasic stroke patients. A randomised controlled trial, *Lancet* **1** (1984), 1197–1200.

[19] C. Mackenzie, An aphasia group intensive efficacy study, *The British Journal of Disorders of Communication* **26** (1991), 275–291.

[20] P.I. Martin, M.A. Naeser, M. Ho, E. Treglia, E. Kaplan, E.H. Baker and A. Pascual-Leone, Research with transcranial magnetic stimulation in the treatment of aphasia, *Current Neurology and Neuroscience Reports* **9** (2009), 451–458.

[21] M. Meinzer, D. Djundja, G. Barthel, T. Elbert and B. Rockstroh, Long-term stability of improved language functions in chronic aphasia after constraint-induced aphasia therapy, *Stroke: A Journal of Cerebral Circulation* **36** (2005), 1462–1466.

[22] M. Meinzer, S. Streiftau and B. Rockstroh, Intensive language training in the rehabilitation of chronic aphasia: efficient training by laypersons,*Journal of the International Neuropsychological Society JINS* **13** (2007), 846–853.

[23] C. Miniussi, S.F. Cappa, L.G. Cohen, A. Flöel, F. Fregni, M.A. Nitsche, M. Oliveri, A. Pascual-Leone, W. Paulus, A. Priori and V. Walsh, Efficacy of repetitive transcranial magnetic stimulation/transcranial direct current stimulation in cognitive neurorehabilitation, *Brain Stimulation* **1** (2008), 326–336.

[24] C.C. Mitchum, M.L. Greenwald and R.S. Berndt, Cognitive treatments of sentence processing disorders: what have we learned? *Neuropsychological Rehabilitation* **10** (2000), 311–336.

[25] A. Moss and M. Nicholas, Language rehabilitation in chronic aphasia and time postonset: a review of single-subject data, *Stroke; a journal of cerebral circulation* **37** (2006), 3043–3051.

[26] M.J. Pickersgill and N.B. Lincoln, Prognostic indicators and the pattern of recovery of communication in aphasic stroke patients, *Journal of Neurology, neurosurgery, and psychiatry* **46** (1983), 130–139.

[27] B.E. Porch, *Porch Index of Communicative Abilities: Therapy and development* **1**, Palo Alto, CA: Psychologists Press, 1967.

[28] F. Pulvermüller, B. Neininger, T. Elbert, B. Mohr, B. Rockstroh, P. Koebbel and E. Taub, Constraint-induced therapy of chronic aphasia after stroke, *Stroke: A Journal of Cerebral Circulation* **32** (2001), 1621–1626.

[29] F. Pulvermüller and M.L. Berthier, Aphasia therapy on a neuroscience basis,*Aphasiology* **22** (2008), 563–599.

[30] R.R. Robey, The efficacy of treatment for aphasic persons: a meta-analysis, *Brain and Language* **47** (1994), 582–608.

[31] R.R. Robey, A meta-analysis of clinical outcomes in the treatment of aphasia, *Journal of Speech, Language, and Hearing Research* **41** (1998), 172–187.

[32] M.L. Rohling, M.E. Faust, B. Beverly and G. Demakis, Effectiveness of cognitive rehabilitation following acquired brain injury: a meta-analytic re-examination of Cicerone et al.'s (2000, 2005) systematic reviews, *Neuropsychology* **23** (2009), 20–39.

[33] R. Schoonen, The internal validity of efficacy studies: design and statistical power in studies of language therapy for aphasics, *Brain and Language* **41** (1991), 446–464.

[34] C.M. Shewan and A. Kertesz, Effects of speech and language treatment on recovery from aphasia, *Brain and Language* **23** (1984), 272–299.

[35] S.L. Small and D.A. Llano, Biological approaches to aphasia treatment, *Current Neurology and Neuroscience Reports* **9** (2009), 443–450.

[36] L.A. Vignolo, Evolution of aphasia and language rehabilitation: a retrospective exploratory study, *Cortex* **1** (1964), 344–367.

[37] R. Whurr, M.P. Lorch and C. Nye, A meta-analysis of studies carried out between 1946 and 1988 concerned with the efficacy of speech and language therapy treatment for aphasic patients, *European Journal of Disorders of Communication* **27** (1992), 1–17.
